# Ethnic disparities in receiving benefits for disability following postpartum mental illness during first two years after delivery: an Israeli nationwide study

**DOI:** 10.1186/s13584-020-00407-z

**Published:** 2020-11-09

**Authors:** Bella Savitsky, Irina Radomislensky, Zhanna Frid, Natalia Gitelson, Saralee Glasser, Tova Hendel

**Affiliations:** 1 Department of Nursing, Ashkelon Academic College, School of Health Sciences, Yitshak Ben Zvi 12, Ashkelon, Israel; 2 Gertner Institute for Epidemiology and Public Health Policy, Sheba Medical Center Tel-Hashomer, Ramat Gan, Israel; 3 The National Insurance Institute of Israel, Research Center, Sderot Weizmann 13, Jerusalem, Israel; 4 Women & Children’s Health Research Unit Gertner, Institute for Epidemiology and Public Health Policy, Sheba Medical Center Tel-Hashomer, Ramat Gan, Israel

**Keywords:** Postpartum mood disturbances, Postpartum depression (PPD), Postpartum mood and anxiety disorders (PMAD), Social security entitlement for benefits, Ethnic groups, Ethnic inequalities

## Abstract

**Background:**

Despite relatively high rates of Postpartum Depression (PPD), little is known about the granting of social security benefits to women who are disabled as a result of PPD or of other postpartum mood and anxiety disorders (PMAD). This study aims to identify populations at risk for underutilization of social security benefits due to PMAD among Israeli women, with a focus on ethnic minorities.

**Methods:**

This retrospective cohort study is based on the National Insurance Institute (NII) database. The study population included a simple 10% random sample of 79,391 female Israeli citizens who gave birth during 2008–2016 (these women delivered a total of 143,871 infants during the study period), and who had not been eligible for NII mental health disability benefits before 2008.

The dependent variable was receipt of Benefit Entitlement (BE) due to mental illness within 2 years following childbirth. Maternal age at delivery, population group, Socio-Economic Status (SES), family status, employment status of the mother and her spouse, and infant mortality were the independent variables. Left truncation COX proportional hazard model with time-dependent variables was used, and birth number served as a time discrete variable.

**Results:**

Bedouin and Arab women had significantly lower likelihood of BE (2.6 times lower and twice lower) compared with other ethnic groups (HR = 0.38; 95% CI: 0.26–0.56; HR = 0.47; 95% CI: 0.37–0.60 respectively). The probability of divorced or widowed women for BE was significantly higher compared to those living with a spouse (HR = 3.64; 95% CI: 2.49–5.33). Lack of employment was associated with higher likelihood of BE (HR = 1.54; 95% CI: 1.30–1.82). Income had a dose-response relationship with BE in multivariable analysis: lower income was associated with the nearly four-fold greater probability compared to the highest income quartile (HR = 3.83; 95% CI: 2.89–5.07).

**Conclusions:**

Despite the exceptionally high prevalence of PMAD among ethnic minorities, Bedouins and Arabs had lowest likelihood of Benefit Entitlement. In addition to developing programs for early identification of postpartum emotional disorders among unprivileged ethnic groups, awareness regarding entitlement to a mental health disability allowance among ethnic minorities should be improved.

## Background

Postpartum mood disturbances are prevalent, being experienced by almost 85% of women after delivery; they generally resolve within a week or two, and are not considered pathological [1]. However, some parturients develop more severe emotional disorders, with postpartum depression (PPD) being the most prevalent [2]. The negative consequences of PPD can impact the mother, the newborn and infant’s physical, emotional, and cognitive development, and the entire family [2, 3]. Globally, the prevalence of PPD has been reported to range from approximately 10–15%, but can be as high as 30% depending on the method and criteria used for determination [4].

The State of Israel had a population of approximately 8,713,300 inhabitants in 2017 [5]. Among the 4,391,500 Israeli females, Jewish and non-Jews (Arabs and others) comprise 79.9 and 20.1% of the population, respectively [5]. These two large major groups include unique sub-populations. For example, immigrants from the Former Soviet Union (FSU) who arrived to Israel during the large immigration wave since 1990 comprise 10% of the population [6]. The Israeli Arab population consists mainly of Muslims, varied by lifestyles, including urban-residents, village-dwellers, and Bedouin who live in encampments or unrecognized townships [7].

An Israeli study, conducted in 2015 among 1128 postpartum women aged 16–48 [8], reported the prevalence of PPD of 10.3%, with the rate being one-third among Jewish women than among Arab women (7 and 21%, respectively) [8]. An earlier study conducted among a sample of 564 Israeli Bedouin women reported an even higher PPD rate of 31% [9]. Among the risk factors found for PPD are lower socio-economic status (SES), low level of education, lack of partner’s support, ethnic minority [1, 8–10], and the birth of an ill child [11].

In Israel the National Insurance Institute (NII) collects social security payment from all employed residents according to their income, and pays benefits according to specific entitlements [12]. The NII provides general disability benefits, which are intended to guarantee persons with disabilities a minimum income for subsistence [13]. The general disability benefit is provided to persons who, due to a physical, mental, or emotional disability resulting from illness, accident, or birth defect; have been diagnosed with a medical disability of at least 40%; are unable to earn a living from employment, or whose earning capacity has been reduced by at least 50% as a result of their disability. Housewives are eligible only if their ability to manage their household has been reduced by at least 50% as a result of disability [14, 15]. The procedure for claiming a NII allowance due to mental health disability begins by submission of relevant medical documents from a family physician, a psychiatrist, or a hospital. The person may then appear before a NII medical committee that must approve existence of mental condition, the level of disability and incapacity.

Despite the relatively high rate of PPD, little is known about the granting of NII disability benefits to women who are unable to work due to PPD or other postnatal emotional disorders. This study aims to describe the phenomenon of social security benefit entitlement (BE) granted due to PMAD among all Israeli female population and to identify groups at risk for underutilization of benefits.

## Methods

### Study population

This is a retrospective cohort study, based on the NII database. The total population included a sample of 778,016 female Israeli citizens who gave birth during 2008–2016 and who had not been eligible for NII mental health disability benefits before 2008. Women who may have been eligible for a benefit based on a physical disability before 2008 were not excluded from the study population. During the study period 1766 women who were diagnosed with postpartum mental illness received BE (Cases). Among the remainder (*n* = 776,250) a simple 10% random sample was drawn (77,625 Controls). Thus, the total study population included 79,391 women, who delivered a total of 143,871 infants during the study period.

### Study variables

*Postpartum-related mental illness*, also referred to as postpartum mood and anxiety disorders (PMAD) included diagnoses of bipolar and depressive disorders, anxiety and stress-related disorders, somatoform and eating disorders (not including psychosis), as per the Diagnostic and Statistical Manual of Mental Disorders, Fifth Edition (REF), which were determined within 2 years following childbirth. Women who received BE due to one of these diagnoses were considered Cases.

*Birth order* was used as the ‘*time*’ variable. Over half of the study population (54.5%) had given birth to their first child during the study period; for 15.7% the study period started from their second child; 14.5% from the third; 7% from their fourth; and for 8.3% the study period started from birth number five or more (Table 1).

**Table 1:** Demographic characteristics of study population at the first delivery during the study period

Demographic characteristics	Cases n (%)	Controls n (%)	Total N (%)
	1766	77,625	79,391
**Age, years; mean (SD)**	28.1 (5.9)	29.2 (5.9)	29.2 (5.9)
**Age Group**			
< 20 years	142 (8.1)	4749 (6.1)	4891 (6.2)
21–40 years	1560 (88.3)	69,247 (89.2)	70,807 (89.2)
40+ years	64 (3.6)	3629 (4.7)	3693 (4.6)
**Population Group**^**a**^			
Israeli-born Jews	1215 (68.8)	46,812 (60.3)	48,027 (60.5)
Arabs (excluding Bedouins)	212 (12.0)	14,541 (18.7)	14,753 (18.6)
Immigrant from Ethiopia	47 (2.7)	1269 (1.6)	1316 (1.7)
Immigrant from FSU	126 (7.1)	6847 (8.8)	6973 (8.8)
Bedouins	52 (2.9)	2800 (3.6)	2852 (3.6)
Other immigrants	112 (6.3)	5327 (6.9)	5439 (6.9)
**Socio-Economic Status**^**a**^**; n (%)**			
I quarter (1–5)	497 (28.2)	18,290 (23.6)	18,787 (23.7)
II quarter (6–9)	594 (33.7)	21,577 (27.8)	22,171 (27.9)
III quarter (10–12)	481 (27.3)	22,194 (28.6)	22,675 (28.6)
IV quarter (13+)	193 (10.9)	15,537 (20.0)	15,730 (19.8)
**Employment status of woman, n (%)**			
Not employed	540 (30.6)	16,673 (21.5)	17,213 (21.7)
Employed	1226 (69.4)	60,952 (78.5)	62,178 (78.3)
Salaried	1184 (67.0)	57,768 (74.4)	58,952 (74.3)
Self-employed	28 (1.6)	2050 (2.6)	2078 (2.6)
Self-employed and salaried	14 (0.8)	1134 (1.5)	1148 (1.4)
**Employment status of spouse, n (%)**			
No spouse	168 (9.5)	3799 (4.9)	3967 (5.0)
Not employed	512 (29.0)	15,523 (20.0)	16,035 (20.2)
Employed	1066 (61.5)	58,303 (75.1)	59,389 (74.8)
Salaried	963 (54.5)	51,041 (65.8)	52,004 (65.5)
Self-employed	83 (4.7)	5070 (6.5)	5153 (6.5)
Self-employed and salaried	40 (2.3)	2192 (2.8)	2232 (2.8)
**Employment status of family, n (%)**			
Nobody employed	261 (14.8)	5590 (7.2)	5851 (7.4)
Somebody Employed	1505 (85.2)	72,035 (92.8)	73,540 (92.6)
**Annual Family Income, NIS; n (%)**			
I quarter (< 49,092)	799 (45.2)	19,391 (25.0)	20,190 (25.4)
II quarter (49,092-107,052)	510 (28.9)	18,633 (24.0)	19,143 (24.1)
III quarter (107,052-199,142)	342 (19.4)	20,336 (26.2)	20,678 (26.0)
IV quarter (< 199,142)	115 (6.5)	19,265 (24.8)	19,380 (24.4)
**Family Status at birth; n (%)**			
Single	147 (8.3)	3403 (4.4)	3550 (4.5)
Married/In stable relationship	1553 (87.9)	72,769 (93.7)	74,322 (93.6)
Divorced/Widow	66 (3.7)	1453 (1.9)	1519 (1.9)
**Birth order of the first index birth in the data set; n (%)**			
1	887 (50.2)	42,348 (54.6)	43,235 (54.5)
2	298 (16.9)	12,182 (15.7)	12,480 (15.7)
3	232 (13.1)	11,277 (14.5)	11,509 (14.5)
4	164 (9.3)	5417 (7.0)	5581 (7.0)
> 5	185 (10.5)	6401 (8.2)	6586 (8.3)

^a^proportion of missing values< 0.5%

*Age* was considered both as a continuous and a categorical variable (< 20 years/21–40 years/40+ years).

*Population group* included Israeli-born Jews, immigrants from the Former Soviet Union (FSU) who had arrived since 1990, immigrants from Ethiopia, Arabs (excluding Bedouins), Bedouins; and Other immigrants (OI) who were not born in Israel.

*Socio-Economic Status (SES)* was defined as the classification of the women’s residence according to the Israel Central Bureau of Statistics Socio-Economic Regional Index, consisting of 20 regional clusters (1 for lowest SES and 20 the highest) [16, 17]. These were categorized into four quarters: I-Quarter SES (1–5), II-Quarter SES (6–9), III-Quarter SES (10–12) and IV-Quarter SES (13–20).

*Family status* of mother at the time of delivery included three categories: married/in a stable relationship, single, and divorced/widowed.

*Employment* The woman’s *employment status* was categorized as having been employed before giving birth as either a salaried worker or freelance. When there was a spouse, the same categorization was made. Women who were not employed during the previous year were defined as housewives. In addition, an variable *employment status of the family* variable was created to designate whether either a woman or her spouse was employed vs. neither employed.

*Infant mortality* was defined dichotomously in cases of the death up to age of 1 year of a child born during the study period.

*Family income* was based on income during the month prior to delivery, and categorized by quartiles.

All variables were treated as time-dependent, except for SES and population group.

### Statistical analysis

The dependent variable was receipt of NII benefit entitlement (BE), defined dichotomously.

**Fig. 1:**
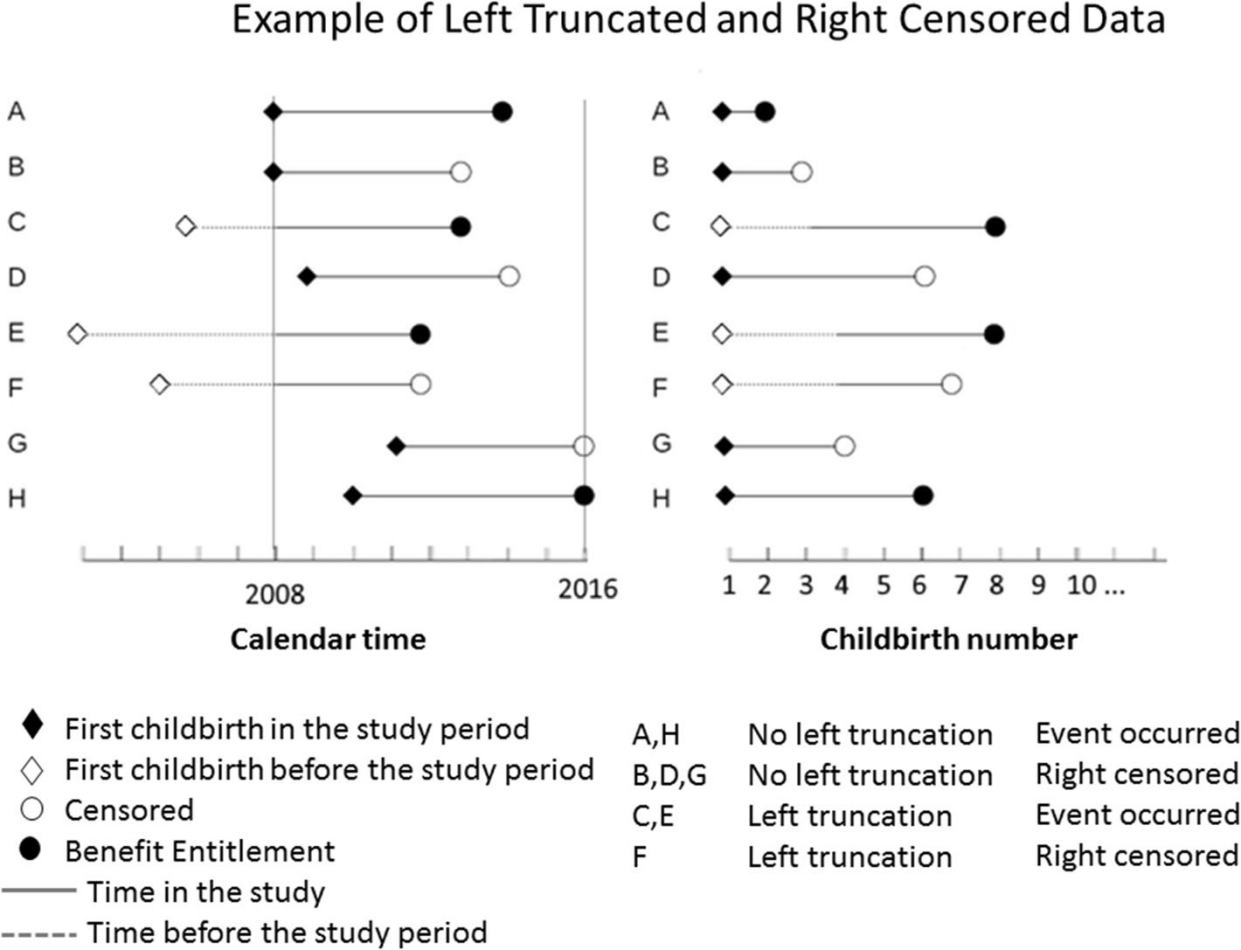
Example of Left Truncated and Right Censored Data

Left truncation COX proportional hazard model with time-dependent variables was used to conduct univariate and multivariable analyses (Fig. 1), with birth number serving as a time-discrete variable. Hazard Ratio (HR) is broadly equivalent to relative risk (RR); useful when the risk is not constant with respect to time. It uses information collected at different times in survival analysis (COX model in this study). Left truncation COX was used, since only for 55% of women was their first delivery included in the study period. SES, family status, employment status and family income were defined as time-dependent variables, while ethnic group was not defined as time dependent.

Further, in order to assess the independent effect of each independent variable, variables found significantly associated with BE in the univariate analysis were included in multivariable analysis.

Before including independent variables in multivariable analysis, correlations between them were checked with Kendall’s Tau coefficient. Most of the correlations were weak, with the strongest reaching Kendall’s Tau coefficient of 0.4. The assumption of proportionality of hazard of each of independent variable was checked and was not violated.

Sensitivity analysis was conducted only among women whose first childbirth occurred during the study period, the results of which (not presented) were consistent with the left truncated model.

The results of all multivariable COX models are presented as adjusted Hazard Ratio (HR) with 95% confidence interval (CI). Statistical analyses were performed with SAS statistical software version 9.2 (SAS Institute, Inc., Cary, NC) and SPSS 25.0 version.

## Results

The study population of 79,391 women, included 1766 Cases received BE on the basis of postpartum mental illness and 77,625 Controls, who did not received mental health disability related BE following delivery. Thus, the overall probability for BE following postpartum mental illness was 2/1000 (**0.2%**) during the 8 years of the study period (after correction for 10% sample for controls).

The demographic characteristics of women who received NII benefits due to postpartum mental illness compared to those who did not are presented in Table 1 (the comparison is made for the first childbirth during the study period). The rate of Israel-born Jews was higher among the Cases (almost 69% vs. 60%) while the rate of ethnic minorities (Bedouins, Arabs, Immigrants from Ethiopia) was lower among the Cases. Cases had lower SES (62% were in the 1–9 social geographic cluster vs. 51% among the Controls) and lower annual family income (45% vs. 25% in the lowest quartile).

Among the Cases 31% of women were unemployed during the year prior to the childbirth, while among the Controls the unemployment rate was 22%.

The proportion of single mothers (single, divorced or widowed) among the Cases was higher than among Controls.

Univariate and multivariable analysis of BE by maternal characteristics are presented in Table 2. Maternal age was not significantly associated BE, thus was not included in multivariable analysis.

**Table 2:** Receipt of NII Benefit Entitlement due to post-partum mental disability, by demographic characteristics (*N* = 79,391)

Demographic characteristics	Univariate		Adjusted^**c**^	
	HR	95% CI	HR	95% CI
**Age, years**	1.00	0.99–1.02	not performed	
**Age Group**				
< 20 years	ref	–	not performed	
21–40 years	1.56	0.69–3.49		
40+ years	1.84	0.77–4.40		
**Population Group**				
Israeli born Jews	ref	–	ref	–
Arabs (excluding Bedouins)	**0.69**	**0.56–0.86**	**0.47**	**0.37–0.60**
Immigrant from Ethiopia	**1.69**	**1.07–2.66**	1.36	0.86–2.16
Immigrant from FSU	**1.27**	**0.95–1.70**	1.07	0.79–1.48
Bedouins	**0.58**	**0.41–0.82**	**0.38**	**0.26–0.56**
Other immigrants	1.01	0.76–1.34	0.86	0.65–1.14
**Socio-Economic Status**				
I quarter (1–5)	**1.47**	**1.14–1.90**	1.19	0.90–1.57
II quarter (6–9)	1.75	1.36–2.25	1.31	1.01–1.71
III quarter (10–12)	**1.51**	**1.16–1.96**	1.26	0.97–1.65
IV quarter (13+)	ref	–	ref	–
**Family Status at birth**				
Single	**3.46**	**2.44–4.89**	**2.33**	**1.62–3.35**
Married/In stable relationship	ref	–	ref	–
Divorced/Widow	**4.28**	**2.95–6.20**	**3.64**	**2.49–5.33**
**Employment status of woman**^**a**^				
Not employed	**1.58**	**1.37–1.82**	**1.54**	**1.30–1.82**
Employed	ref	–	ref	–
**Employment status of spouse**^**b**^				
No spouse	**3.63**	**2.63–4.99**	**1.80**	**1.27–2.54**
Not employed	**1.20**	**1.02–1.39**	**0.60**	**0.50–0.72**
Employed	ref	–	ref	–
**Employment status of family** ^**b**^				
Nobody employed	**1.73**	**1.43–2.11**	1.21	0.97–1.52
Somebody Employed	ref	–	ref	–
**Infant mortality (to 1 year)**				
Yes	2.16	0.97–4.81	not performed	
No	ref	–		
**Annual Family Income. NIS**				
I quarter (< 49,092)	**4.28**	**3.31–5.52**	**3.83**	**2.89–5.07**
II quarter (49,092-107,052)	**3.47**	**2.68–4.50**	**3.49**	**2.66–4.58**
III quarter (107,052-199,142)	**2.55**	**1.94–3.36**	**2.54**	**1.93–3.35**
IV quarter (199,142+)	ref	–	ref	–

^a^ employment status of woman, employment status of spouse, employment status of any family member were not included together in the same model, but in the separate models

^b^ multivariable model with employment status of spouse did not include family status of woman

^c^ multivariable model adjusted for population group, SES, family status, employment (of women, spouse or any family member) and income

Bedouins and Arabs had significantly lower probability of BE, and this association remained significant after adjustment for other variables: the likelihood of Bedouins to receive benefits was 2.6 times lower and of Arabs twice lower than that of other population groups (HR = 0.38; 95% CI: 0.26–0.56; HR = 0.47; 95% CI: 0.37–0.60 respectively). Upon univariate analysis, immigrants from Ethiopia were more likely to receive benefits, however this advantage disappeared when adjusting for the other variables. Geographical SES was also associated with BE in the univariate model, but was no longer associated after adjusting for other characteristics.

The likelihood of single, divorced, or widowed women to receive benefits was significantly higher than that for married women, even after adjustment, with divorced/widowed women most likely to receive benefits (HR = 3.64; 95% CI: 2.49–5.33).

Upon univariate analysis, unemployed women had the greatest probability of receiving benefits and this association remained unchanged after adjustment in the multivariable analysis (HR = 1.54; 95% CI: 1.30–1.82). When the woman’s employment status was replaced in the model by any family member occupation, no association was found in multivariable analysis.

Spouse’s unemployment was also associated with higher likelihood of BE (HR = 1.20; 95% CI: 1.029-1.39), but after adjustment for income, the association changed; thus wives whose spouse was unemployed had a 67% lower probability of BE (HR = 0.60; 95% CI: 0.50–0.72).

Family income had an inverse dose-response relationship with BE in multivariable analysis: the lower the income, the higher was the likelihood to receive benefits. Those in the lowest income quartile were almost four times as likely to receive BE, compared to those in the highest quartile (HR = 3.83; 95% CI: 2.89–5.07).

## Discussion

The findings of this study point to very low rate of NII benefit entitlement (BE) due to postpartum mental illness. The most prevalent postpartum emotional disorder is postpartum depression (PPD), and although studies have reported approximately a 10% prevalence in the Israeli population [18], the overall likelihood of BE pursuant to postpartum mental illness was only 0.2% during the 8 years of the study period. Thus, the likelihood of BE in cases in which the women are suffering from PPD is significantly lower than risk of PPD itself, particularly among certain population groups, among whom even higher PPD rates have been reported.

In the study, which includes data from all Mother and Child Healthcare Clinics in Israel, the incidence of postpartum depression is 5% [19]. Given the annual number of births in Israel (180,000) [20], it is expected that 9000 new cases of postpartum depression are diagnosed annually, of which half of them (4500) are severe [19]. Our study points to 220 annual cases of benefit entitlement following mental illness, which is clearly much lower than expected.

While increased rates of PPD have been reported among Israeli Bedouins and Arabs, compared to Israeli Jews [7, 9, 21, 22], their probability of BE for postpartum mental health disability was significantly lower. In general, NII claims in cases of mental illness among the Arab minority is significantly lower than among the Jewish population [23]. In addition to recognized barriers that make it difficult to realize the mental health disability benefits, such as fear of stigmatization, language barriers, and lack of knowledge, there are unique barriers in Arab society associated with cultural perceptions of people with mental disabilities [24].

A major factor influencing claiming of benefits is awareness that such rights exist, and knowledge of the eligibility rules and claiming procedures [25]. A gap has been found between Jews and Arabs regarding knowledge and understanding of health insurance [26], and this may also be the case regarding NII benefit eligibility rights. In the case of conditions such as PPD, women in ethnic minority groups may also be less cognizant of the fact that they are suffering from a postpartum emotional disorder [27]. In order to file a claim for NII benefits, a woman must present medical documents, and since the percentage of undiagnosed disorders is likely to be high in this population [28], this presents an additional barrier for claiming of rights in case of PPD. The 2015 Israeli State Comptroller Report highlighted the lack of maximizing insurance rights, and recommended that social workers in welfare departments or in the health care system should serve as a bridge between citizens who do not know their rights and government officials [29]. The contribution of social workers is crucial especially among unprivileged groups.

A second factor hindering the opportunity to claim a benefit is related to bureaucratic complications, including the need to arrive at the appropriate government office, complete necessary forms, presenting medical documents from primary care physician or hospital, and the requirement to appear before a medical committee. For example, although Israeli Bedouins, who live in recently established permanent settlements (60%) or in a traditional tribal settlements (40%) [30], are eligible for NII disability benefits, many Bedouin women cannot get to government offices due to lack of transportation from their villages [31]. In addition, language may also be a barrier, since most Bedouin women (80%) are illiterate [31, 32]. Thus, despite the fact that NII forms are available in Arabic, this would not be sufficient for illiterate women to complete them without assistance.

Psychological and cultural beliefs may prevent people from claiming benefit rights due to fear of being labeled as “ill,” “weak,” or being a “cheater” [24]. This may influence all ethnic groups, since tagging someone as mentally ill may interfere with future possibilities, such as employment. Further, most people experiencing mental health problems do not seek professional help, and the stigma of mental illness is considered a major barrier to seeking appropriate treatment [26]. A recent study conducted in Israel found that only 76% of women participated in a screening initiative for PPD and participation was associated with more positive attitudes toward seeking help [33].

Unemployed women were significantly more likely to claim BE. Employment in itself is considered helpful to people with mental disorders [34], and in this study a women’s unemployed status prior to childbirth was associated with receiving benefits, even after adjustment for total family income. This association may be related to higher incidence of PPD among unemployed women, which may be due greater social isolation and increased childcare responsibilities [35, 36].

Interestingly, although the rate of unemployment was found to be a predictor of receiving BE, the rate of unemployment among Arab women in general is very high [37, 38], in this study population (71% among Bedouin and 53% among Arab women), while the probability of their receiving BE was lower. Thus, it is of note that those women who might be more likely to qualify for the benefits, and who have higher rates of perinatal emotional disorders, are possibly underutilizing their rights. Indeed, a study conducted among Bedouin women [27], reported multiple barriers that interact to limit Bedouin women’s access to PPD treatment, including lack of culturally appropriate health-care services, lack of PPD screening and detection procedures; stigmatization of mental health problems, as well as at the public policy level: residence in unrecognized villages lacking basic infrastructure [27].

Single, divorced, and widowed women were more likely to receive BE, compared to those with a spouse. This may reflect the higher incidence of PPD which has been reported among women without partner support [39], as marital status was associated with BE independently from income.

Income was negatively associated with BE, i.e., the rate was increasingly higher in the lower income groups, compared to the highest. Low income has been found to be a risk factor for a low level of awareness of the need for mental health treatment, leading to not seeking professional help [40]. Further, low SES likely exposes women to increased stress, which is also a risk factor for PPD. [39, 41] Those in the lower income groups likely have a greater incentive to claim BE than do those in the higher income group, and families with higher incomes can more easily manage financially even if the woman is unable to work due to mental health disability. Income served as a mediator in the association between employment of any family member and likelihood of BE; unemployment of either was significantly associated with BE, but after inclusion of income in the model, the association disappeared.

In the present study infant mortality was associated with a two times greater probability of BE. Although the association was not statistically significant, the strength of the association suggests that the finding may be valid. According to the World Health Survey, conducted among 59,444 women, women who experienced a child’s death had higher odds for all types of mental illness and depression (OR = 1.64; 95% CI:1.39–1.93) [42]. The results of the present study indicate that despite higher odds of developing depression, the death of an infant does not independently influence claiming BE. In contrast, it is clear that in the case of death of a infant, it is crucial that the process of claiming benefits coincide with support, surveillance, guidance and assistance.

### Limitations

Despite the broad scope of the NII database, it posed certain limitations. With respect to family income, it included only income as reported to the Israeli Tax Authority. It has been found that there is a gap between reported income in surveys and income reported to the Israeli Tax Authority; this gap reaches 26% in the Arab population, compared to other groups [43]. The family status variable can also pose a problem since approximately 20–40% of Bedouin families are polygamic, and in most cases second and third wives are not registered as ‘married’ in the NII database. In these cases, the women are not registered as married, thus their spouse’s income cannot be estimated. Other potentially interesting aspects of the study topic, such as the rate of BE by ethnic group or level of religiosity, are also not currently available on the NII database. Future research may be able to access this information.

For the purpose of this research, the decision to attribute the mental health disability to the childbirth within the preceding 2 years (rather than the more commonly accepted 1 year time span) and label it as “postpartum” was made upon the recommendation of the NII Disability Department, based on their experience regarding the length time required for completing all stages of the claim process. Thus, it is possible that the condition was not related to the childbirth but nevertheless was defined in this study as “case” if it occurred. Therefore, the rate of false positives could not be assessed.

### Strengths

A strength of this retrospective cohort study is that it is based on a simple random sample of all Israeli residents, therefore sampling bias would not affect the results. In addition, the usage of left-truncated Cox model made it possible to correct the analysis for the fact that childbirths occurred before 2008 were not included in the study period.

### Policy implications and recommendations

In Israel Mother-Child Healthcare Clinics (MCHC) conduct the child’s health and developmental follow-up during the first years of life, including vaccinations in accordance with the Ministry of Health’s immunization program. In addition, since 2013, a universal PPD screening program using the Edinburgh Postnatal Depression Scale (EPDS) [44] has been implemented in these clinics, providing an opportunity for early detection of PPD symptoms [45]. While the EPDS is a written self-rating questionnaire, and there is an Arabic-language translation, some women, particularly Bedouin, are analphabetic. Guidelines should be prepared for screening these women appropriately, thereby improving the possibility of PPD detection in these ethnic groups.

Social workers should work with MCHC nurses, and when cases of severe PPD are detected, in addition to appropriate intervention and referral, they should raise women’s awareness regarding entitlements to NII disability benefits for women suffering postpartum emotional disorders. In cases where no social worker is available, nurses should be able to provide the relevant information. This is true for all women, and particularly for those in ethnic minorities, and for families in which the woman is unemployed.

Another opportunity to reach women who may be entitled to disability benefits because of PPD is at HMO clinics, which they may visit following delivery. Family physicians, gynecologists, as well as pediatricians and nurses, may become aware of the women’s emotional distress or disorder, and they should be equipped to offer relevant information to women who may be eligible for benefits, or to refer them to HMO social worker for guidance.

Further, assistance in applying for benefits should be offered by the NII to women who experience difficulties in the application process itself. Beyond the availability of Arabic-language forms, efforts should also be made to ensure that women suffering from postpartum emotional disorders are referred to social workers at NII offices or to the organization “Guided Hand” [46], which offers assistance in applying for benefits claims for disability.

### What is already known about this subject

- Postpartum mood disturbances are frequent, being experienced by almost 85% of women after delivery.
- According to the most recent evidence, prevalence of Postpartum depression in Israel is 10.3% (7% among the Jewish women, 21% among the Arab women and 31% among the Bedouin women).
- Despite the high rates of Post-Partum Depression, little is known about the granting of social security benefits to women who are disabled as a result of this or other postpartum mental disorders.

### What this study adds

- Despite higher prevalence of Postpartum Depression among ethnic minorities, Bedouins and Arabs had significantly lower likelihood of Benefits Entitlement (2.6 times lower and twice lower than that of other ethnic groups respectively).
- The likelihood of divorced or widowed women and of women who were unemployed prior to childbirth, to receive Benefit Entitlement was significantly higher compared to those living with a spouse and compared to employed women.
- Income had a dose-response relationship with Benefit Entitlement, while lower income quartile being associated with a nearly four-fold greater probability for BE compared to the highest income quartile.
- Efforts should also be made to ensure that women suffering from postpartum emotional disorders be referred to social workers, who can help them claim their disability rights.

## Data Availability

Data sharing is not applicable to this article, as the research center of the National Insurance Institute of Israel does not allow any files, even those that are de-identified, to be released.
